# Infectious disease testing of UK-bound refugees: a population-based, cross-sectional study

**DOI:** 10.1186/s12916-018-1125-4

**Published:** 2018-08-28

**Authors:** Alison F. Crawshaw, Manish Pareek, John Were, Steffen Schillinger, Olga Gorbacheva, Kolitha P. Wickramage, Sema Mandal, Valerie Delpech, Noel Gill, Hilary Kirkbride, Dominik Zenner

**Affiliations:** 1 Travel and Migrant Health Section, National Infection Service, Public Health England, 61 Colindale Ave, London, NW9 5EQ UK; 2 Department of Infection, Immunity and Inflammation, University of Leicester, Leicester, UK; 3 International Organization for Migration (IOM), Citibank Center, 28th Floor, 8741, Paseo de Roxas, Makati, 1200 Metro Manila, Philippines; 4 International Organization for Migration (IOM), 17 Route des Morillons, 1218 Grand-Saconnex, Switzerland; 5 Immunisation, Hepatitis and Blood Safety, National Infection Service, Public Health England, 61 Colindale Ave, London, NW9 5EQ UK; 6 HIV and STI Department, National Infection Service, Public Health England, 61 Colindale Ave, London, NW9 5EQ UK; 7 TB Screening Unit, National Infection Service, Public Health England, 61 Colindale Avenue, London, NW9 5EQ UK; 8 Institute for Global Health, Faculty of Population Health Sciences, University College London, Gower Street, London, WC1E 6BT UK

**Keywords:** Refugees, Refugee health, Health assessment, Infectious diseases, Migrant health

## Abstract

**Background:**

The UK, like a number of other countries, has a refugee resettlement programme. External factors, such as higher prevalence of infectious diseases in the country of origin and circumstances of travel, are likely to increase the infectious disease risk of refugees, but published data is scarce. The International Organization for Migration carries out and collates data on standardised pre-entry health assessments (HA), including testing for infectious diseases, on all UK refugee applicants as part of the resettlement programme. From this data, we report the yield of selected infectious diseases (tuberculosis (TB), HIV, syphilis, hepatitis B and hepatitis C) and key risk factors with the aim of informing public health policy.

**Methods:**

We examined a large cohort of refugees (*n* = 18,418) who underwent a comprehensive pre-entry HA between March 2013 and August 2017. We calculated yields of infectious diseases stratified by nationality and compared these with published (mostly WHO) estimates. We assessed factors associated with case positivity in univariable and multivariable logistic regression analysis.

**Results:**

The number of refugees included in the analysis varied by disease (range 8506–9759). Overall yields were notably high for hepatitis B (188 cases; 2.04%, 95% CI 1.77–2.35%), while yields were below 1% for active TB (9 cases; 92 per 100,000, 48–177), HIV (31 cases; 0.4%, 0.3–0.5%), syphilis (23 cases; 0.24%, 0.15–0.36%) and hepatitis C (38 cases; 0.41%, 0.30–0.57%), and varied widely by nationality. In multivariable analysis, sub-Saharan African nationality was a risk factor for several infections (HIV: OR 51.72, 20.67–129.39; syphilis: OR 4.24, 1.21–24.82; hepatitis B: OR 4.37, 2.91–6.41). Hepatitis B (OR 2.23, 1.05–4.76) and hepatitis C (OR 5.19, 1.70–15.88) were associated with history of blood transfusion. Syphilis (OR 3.27, 1.07–9.95) was associated with history of torture, whereas HIV (OR 1521.54, 342.76–6754.23) and hepatitis B (OR 7.65, 2.33–25.18) were associated with sexually transmitted infection. Syphilis was associated with HIV (OR 10.27, 1.30–81.40).

**Conclusions:**

Testing refugees in an overseas setting through a systematic HA identified patients with a range of infectious diseases. Our results reflect similar patterns found in other programmes and indicate that the yields for infectious diseases vary by region and nationality. This information may help in designing a more targeted approach to testing, which has already started in the UK programme. Further work is needed to refine how best to identify infections in refugees, taking these factors into account.

**Electronic supplementary material:**

The online version of this article (10.1186/s12916-018-1125-4) contains supplementary material, which is available to authorized users.

## Background

International migration has increased significantly (by 41%) since 2000. In 2015, it was estimated that there were 244 million international migrants globally, the majority (151 million) with destination countries in Europe and Asia [1]. In many recipient countries, international migration is becoming an increasingly important determinant of population change. For instance, in January 2016, it was estimated that 35 million residents (approximately 6.9% of the European Union (EU) population) in the EU were born outside of the EU, in addition to 19.3 million persons who were living in a different EU Member State from the one in which they were born [2, 3]. Forcible displacement, as a result of conflict, persecution, violence or human rights violations, has also reached a record-high, with an estimated 21.3 million refugees globally in 2015; an increase of 55% since year-end 2001. This is largely attributable to the ongoing civil conflict in the Syrian Arab Republic [4].

A number of countries have official resettlement programmes for refugees, including the USA, Canada, Australia, New Zealand, the UK and many others [5–7]. The UK government accepts refugees under four different schemes, namely the Gateway Protection Programme, the Mandate Resettlement Scheme, the Syrian Vulnerable Persons Resettlement Scheme (VPRS) and the Vulnerable Children Resettlement Scheme (VCRS) (hereafter collectively the ‘UK programme’). The Gateway Protection Programme has committed to resettle approximately 750 refugees per year on the basis of their refugee status and need for resettlement [8]. The Mandate Resettlement Scheme is much smaller, and applicable only to individuals who have been granted refugee status by the United Nations High Commissioner for Refugees (UNHCR) and who have close ties^1^ to the UK. The VPRS and VCRS, on the other hand, represent specific resettlement schemes that the UK has devised to offer protection to people on a larger scale in times of crisis [9]. For this reason, and due to its recent rapid expansion, the VPRS is probably the most high-profile of the UK schemes.

The VPRS was established by the UK Government in January 2014 in response to the Syrian crisis [10]. It aims to enable vulnerable Syrians and other nationalities affected by the conflict to settle in the UK, prioritising those who meet the UNHCR vulnerability criteria, including women and children at risk, survivors of violence or torture, refugees with legal or physical protection needs, medical needs or disabilities, children and adolescents at risk, and refugees with family links in resettlement countries [10]. Initially small, and with no fixed quota, it has increased in prominence following a pledge by the UK Government in September 2015 to resettle up to 20,000 people from the Syrian region by 2020 [11]. This has attracted heightened media coverage and public interest (Additional file 1: Appendix III).^2^ Subsequently, the VCRS was established in January 2016 to support and resettle up to 3000 vulnerable and refugee children and their families affected by the conflict [12]. As of the last quarter of 2016, 20,878 refugees had been resettled through the entire UK programme [13].

Under these schemes,^3^ refugees are referred by the UNHCR and reviewed by UK authorities for resettlement in the UK. Prior to departure, a detailed health assessment (HA) is performed by the International Organization for Migration (IOM). The aim of the HA is to facilitate early integration of the refugee, promoting individual health, protecting public health where relevant and linking individual needs with appropriate health and social services in the UK. The UK HA protocol has recently been reviewed and updated with this in mind, to align it more closely with UK public health policy and best practice [14]. The components of the HA are briefly outlined in Table 1.

**Table 1:** Components of the standardised pre-entry health assessment for refugee applicants

Standardised pre-entry health assessment components	
General assessment	Medical history
	Physical examination (vital signs, assessment of systems, oral and dental examination, skin examination, developmental milestones for children)
	Routine laboratory and radiological examinations, including urinalysis and chest x-ray
Testing for specific conditions	Tuberculosis (according to the UK tuberculosis technical instructions [38]) HIV Syphilis Other sexually transmitted infections Hepatitis B and C Helminthic infection (as appropriate, according to protocol) Malaria (as appropriate, according to protocol)
Immunisation	According to the UK immunisation schedule [39]
Additional clinical assessments	Relating to other chronic, physical, psychosocial or mental health issues, as appropriate

There is evidence that most migrants in Europe, at least initially, are relatively healthy compared to the host population, although migrants do face specific health challenges and may experience a deterioration in health over time in the host country [15, 16]. It is possible that refugees, including those resettled through international resettlement schemes, may be at slightly higher risk of infectious diseases due to a higher prevalence of these diseases in their country of origin, specific circumstances of their residency and travel, and programme selection criteria which favour vulnerable migrants. However, there is limited information available on the exact epidemiology of infectious diseases in these groups. Therefore, there is a need to analyse these data and compare them with other sources of prevalence figures to ensure that appropriate public health measures, including HA, can be applied to these population groups most at risk and that individuals can be thus linked early to appropriate healthcare services in the UK.

This paper aims to analyse and describe, for the first time, data on the prevalence of all infectious diseases (tuberculosis (TB), HIV, syphilis, hepatitis B and hepatitis C) from a large cohort of refugees who underwent comprehensive pre-entry health assessments as part of the UK resettlement programme. It compares the recorded prevalence against published estimates in order to assess whether moving to risk-based testing would be feasible.

## Methods

### Study design, participants and consent

**Fig. 1:**
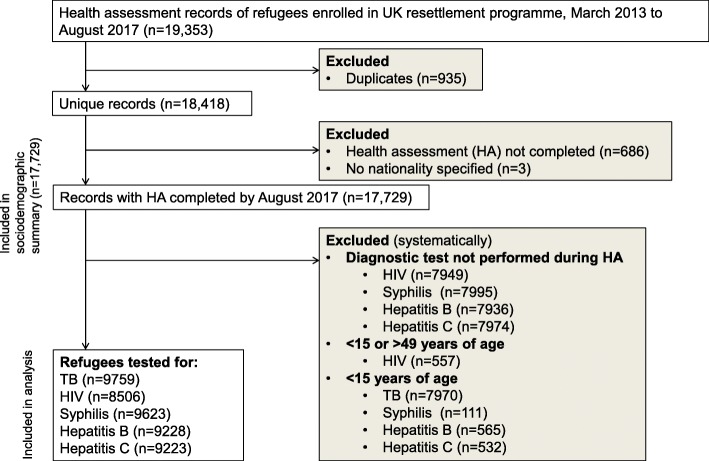
Flow diagram illustrating selection criteria used to identify the study sample

We undertook a population-based cross-sectional study of all refugees included in the UK programme (*n* = 18,418) who had a complete HA conducted by IOM between March 2013 and August 2017. Applicants whose HA was not completed were excluded (*n* = 686). Additional exclusion criteria were applied during analysis (Fig. 1). In general, the subjects included versus those excluded were similar in their demographic characteristics (Additional file 1: Appendix IV). The reporting of this study conforms to the STROBE statement (Additional file 1: Appendix V). As part of the testing process, applicants consented for their data to be used by the relevant UK authorities and agencies.

### Data sources

Data were collected from all 22 IOM clinics enrolled in the UK pre-entry migration HAs in 14 countries, according to a standardised pro forma.^4^ Laboratory/radiology services were performed by IOM or contracted providers where local clinic capacity did not permit carrying out these services in house.

Data were entered directly into the electronic form by the examining physician/nurse at the time of examination, and any additional hand-written notes incorporated into qualitative fields. All information was entered into the medical module of IOM’s electronic database system, the Migrant Management Operational System Application (MiMOSA), which has a set of data validation rules in place, and further data validation was done by the IOM medical department using statistical and database functions. Data was saved as a transactional database using the Microsoft SQL Server.

Data were extracted for the current study on demographics (sex, age, nationality, country of examination, position in family) and infectious disease testing results (HIV serology, syphilis testing, other sexually transmitted infection (STI) testing, TB chest x-ray, TB clinical signs and symptoms, TB culture, TB smear, hepatitis B serology (hepatitis B surface antigen and any additional markers) and hepatitis C serology (hepatitis C antibody, anti-hepatitis C antibody, and hepatitis C virus RNA)). All cases were classified using pre-defined case definitions and further corroborated against the physician’s notes and/or laboratory notes to ensure rigour. Active TB cases were identified in a two-step process. First, suspected cases were identified from clinical and radiological database variables. These were then individually verified by each IOM clinic and categorised as active TB on the basis of culture confirmation. Further information on testing cohorts and case definitions are included in Additional file 1: Appendix I.

Country-specific prevalence estimates for the infectious diseases of interest were also extracted from annual World Health Organization (WHO) country reports and/or the literature [17–22].

### Data management and statistical analysis

Data cleaning and analyses were carried out using Stata version 13.1 [23]. All tests were two-tailed and *p* values less than 0.05 were regarded as significant. A full description of the data management, variable classifications and definitions is provided in Additional file 1: Appendix I.

Briefly, data analysis was undertaken in several steps. We first described the demographics of applicants tested, and summarised continuous data with median and interquartile range and described categorical responses as a simple descriptive percentage, with (95% confidence interval (CI)), and comparisons made using Pearson’s χ^2^ test.

For each of the infectious diseases of interest we calculated the absolute numbers of positive test results, the proportion positive (number of individuals testing positive divided by the number of eligible applicants tested; this was the testing yield or positivity rate of the individual diseases in the cohort), stratified by nationality.

We calculated testing yield of the different diseases, stratified by nationality, and presented these next to published disease-specific country level prevalence rates.

Univariable and multivariable logistic regression analyses were conducted to assess factors associated with case positivity. The model was built in stepwise forward fashion evaluating each variable for inclusion using likelihood ratio tests. Age, sex, world region of nationality, exam year and history of displacement were adjusted for in each multivariable model as well as additional variables specific to each outcome (Additional file 1: Appendix II). Interaction was only tested where biologically plausible. Certain variables were removed from the final model to reduce collinearity (Additional file 2: Tables S1–S5). Cluster analysis was performed to account for correlation that may occur between individuals of the same immediate family, based on their resettlement case number.^5^ For TB, we restricted all analyses to confirmed cases of active TB, but repeated multivariable analysis with suspected cases (Additional file 1: Appendix VI). The limited number of events in the confirmed case analysis limits statistical certainty for that analysis.

## Results

### Demographics of cohort

Between March 2013 and August 2017, 18,418 applicants for resettlement in the UK were screened by IOM in clinics in 14 different countries. Of these, 17,729 (96.3%) applicants had undergone at least one complete pre-entry HA at the time of data extraction (August 2017) and were included in the analysis. The majority of applicants (16,055, 90.6%) were nationals of the WHO Eastern Mediterranean Region^6^ and the African Region^7^ (AFR; 1608, 9.1%), representing 29 countries. Just over half were male (51.2%) and median age was 18 years (interquartile range 7–33 years). There were 4665 (26.3%) principal applicants,^8^ whilst the majority (12,943, 73.0%) of applicants were their family or dependents (defined as immediate family, i.e. spouse/civil partner, children, parents/step-parents, siblings). The mean family size was estimated at 3.8 persons.

### Infectious disease yield and exposure factors identified

The number of refugees included in the yield calculation and logistic regression analysis varied by disease and ranged from 8506 to 9759 (Fig. 1). Of the five infectious diseases of interest, the most commonly identified infections were hepatitis B (188 cases out of 9228 tested). Relatively fewer cases of hepatitis C (38/9223), HIV (35/8506), syphilis (23/9623) and active TB (9/9759) were identified.

The magnitude of overall testing yields for hepatitis B (2.04%, 95 % CI 1.77–2.35%) were particularly high. Testing yields for the other infections remained under 1.0% but varied widely by nationality.

A total of 4 applicants with coinfections were identified: HIV-syphilis (*n* = 2) and HIV-hepatitis B (*n* = 2). No applicant had more than 2 concurrent infections.

### Active TB

Of 9 active TB cases, 6 (67%) were male and 7 (78%) were aged 25–49. Cases came from the Democratic Republic of Congo (DRC), Ethiopia, Somalia and Syria. The total testing yield for active TB was 92 (95% CI 48–177) cases per 100,000 but varied widely by nationality from 42 (13–129) per 100,000 for Syria to 526 (170–1621) per 100,000 for DRC. The testing yields in this study were relatively consistent with WHO prevalence rates (last available data 2014) for those nationalities with positive cases; however, a number of nationalities of countries with high TB prevalence also yielded zero positive cases, mostly because of low screening throughput (Table 2).

**Table 2:** Active tuberculosis (TB) yield per 100,000 population among tested applicants compared to WHO country TB prevalence estimates per 100,000 population (reference year 2014), by country of nationality

Country of nationality	Number screened (n)	Number of cases detected (%)	TB yield per 100,000 among tested applicants (95% CI)^a^	WHO country prevalence per 100,000 (95% CI), 2014 reference year [22]
Afghanistan	63	0	0	340 (178–555)
Democratic Republic of Congo	570	3 (0.53)	526 (170–1621)	532 (282–859)
Eritrea	59	0	0	123 (63–203)
Ethiopia	290	1 (0.34)	345 (48–2414)	200 (161–243)
Iran	15	0	0	33 (17–55)
Iraq	540	0	0	67 (35–111)
Palestine	28	0	0	N/A
Somalia	562	2 (0.36)	356 (89–1413)	491 (254–805)
South Sudan	40	0	0	319 (139–572)
Sudan	369	0	0	151 (67–267)
Syria	7195	3 (0.04)	41 (13–129)	19 (6.2–39)
Uganda	2	0	0	159 (87–253)
Other AFR^b^	8	0	0	
Other EMR^c^	9	0	0	
Other EUR^d^	5	0	0	
Other^e^	4	0	0	
Total	9759	9 (0.09)	92 (48–177)	

^a^TB yield was calculated on adults aged > 15 for ethical reasons and consistency

^b^Other AFR included Burundi, Congo, Rwanda, Cameroon, Nigeria

^c^Other EMR included Jordan, Lebanon, Djibouti, Yemen, Pakistan

^d^Other EUR included UK, St Helena, Switzerland, Turkey

^e^Other included Solomon Islands, China, Taiwan or applicants with no nationality specified

*CI* confidence interval

Additional file 2: Table S1 presents details of the univariable and multivariable regression analyses for active TB (*n* = 9). On multivariable analysis, the adjusted odds of active TB remained significantly higher for applicants who had a past history of TB infection (adjusted odds ratio (aOR) 145.53, 95% CI 25.99–814.84, *p* < 0.001) after adjusting for age, sex, WHO region of nationality, year of examination and history of displacement. The confirmed case analysis was limited by the low number of events for some variables, so we carried out an additional analysis with suspected cases (*n* = 134) (Additional file 1: Appendix VI). This showed similar findings, albeit with slightly changed effect sizes (notably, aORs of suspected TB were significantly higher with increasing age and among applicants who were examined in 2014, had past history of TB and had a household member with history of TB).

### HIV

Of 35 HIV cases, 7 (20%) were male and 31 (89%) cases were aged between 15 and 49 years. The overall HIV positivity rate among adults aged 15–49 years was 0.36% (0.25%–0.50%). The rate ranged by nationality, from 0.6% (0.2%–1.8%) among nationals from Somalia to 3.6% (2.3%–5.6%) among nationals from the DRC. Compared to WHO prevalence estimates, rates were generally higher (by up to 5 times among DRC nationals) (Table 3).

**Table 3:** HIV yield (%)^a^ among tested applicants aged 15–49 years, compared to WHO country HIV prevalence estimates (%) in adults aged 15–49 years (reference year 2016), by nationality

Country of nationality	Number screened (n)	Number of cases detected (%)	HIV positivity rate in 15–49 year olds in tested cohort, % (95% CI	Estimated country prevalence, 15–49 year olds, 2016, % (95% CI) [18]
Afghanistan	56	0	0.0	< 0.1 (< 0.1 to < 0.1)
Democratic Republic of Congo	504	18 (3.57)	3.6 (2.3–5.6)	0.7 (0.6–0.9)
Eritrea	52	0	0.0	0.6 (0.4–0.9)
Ethiopia	259	4 (1.54)	1.5 (0.6–4.0)	1.1 (0.8–1.3)
Iran	14	0	0.0	0.1 (< 0.1–0.2)
Iraq	462	1	0.0	No data
Palestine	25	0	0.0	No data
Somalia	499	3 (0.60)	0.6 (0.2–1.8)	0.4 (0.2–0.5)
South Sudan	35	0	0.0	2.7 (1.7–4.0)
Sudan	329	5 (1.52)	1.5 (0.6–3.6)	0.2 (0.1–0.4)
Syria	6245	0	0.0	No data
Uganda	1	0	0.0	6.5 (6.1–7.0)
Other AFR^b^	8	0	0.0	4.2 (3.7–4.8)^f^
Other EMR^c^	8	0	0.0	0.1 (< 0.1–0.2)^f^
Other EUR^d^	4	0	0.0	0.4 (0.4–0.4)^f^
Other WPR^e^	4	0	0.0	0.1 (< 0.1–0.2)
Total	8506	31 (0.36)	0.4 (0.3–0.5)	

^a^ HIV yield was calculated on adults aged 15–49 for ethical reasons and for ease of comparison to reference ranges from WHO

^b^Other AFR included Burundi, Congo, Rwanda, Cameroon, Nigeria

^c^Other EMR included Jordan, Lebanon, Djibouti, Yemen, Pakistan

^d^Other EUR included UK, St Helena, Switzerland, Turkey

^e^Other WPR included Solomon Islands, China, Taiwan or applicants with no nationality specified

^f^Regional prevalence comparisons for AFR, EMR, EUR and WPR are based on estimates from WHO Member States

*CI* confidence interval

On multivariable analysis, those who remained with significantly higher odds of being HIV positive included women from the AFR region (aOR 51.72, 95% CI 20.67–129.39, *p* < 0.001), aged 35–49 (5.76, 2.05–16.22, *p* = 0.001) and with past history of STI (aOR 1521.54, 342.76–6754.23, *p* < 0.001). Those who remained with significantly lower odds of HIV included males (0.18, 0.07–0.50, *p* = 0.001) who were examined in 2014–2016 (2014: 0.11, 0.02–0.55, *p* = 0.007; 2015: 0.28, 0.08–0.97, *p* = 0.043; 2016: 0.35, 0.13–0.97, *p* = 0.043) (Additional file 2: Table S2).

### Syphilis

Of 23 cases, 14 (61%) were male and 18 (78%) were aged between 15 and 49 years. The overall syphilis testing yield among adults aged 15 years and older was 0.24% (0.15–0.36%). The lowest non-zero yield was among Syrian nationals at 0.06% (0.02–0.15%) and the highest yield 3.33% (1.90–5.78%) among Sudanese nationals. Yields were generally lower in the screened cohort compared to WHO country prevalence estimates (Table 4).

**Table 4:** Syphilis yield (%)^a^ in tested applicants ≥15 years of age compared to WHO syphilis seropositivity among antenatal care attendees, by country of nationality (reference year 2015 unless otherwise stated)

Country of nationality	Number screened (n)	Number of cases detected (%)	Yield in tested cohort, % (95% CI)	Syphilis seropositivity among antenatal care attendees, 2015, % [17]^b^
Afghanistan	60	0	0.0	0.6
Democratic Republic of Congo	570	2 (0.35)	0.35 (0.09–1.39)	1.9
Eritrea	55	0	0.0	0.6
Ethiopia	277	3 (1.08)	1.08 (0.35–3.31)	1.1
Iran	15	0	0.0	0.0^g^
Iraq	538	1 (0.19)	0.19 (0.03–1.31)	0.0^h^
Palestine	28	0	0.0	N/A
Somalia	554	0	0.0	5.9
South Sudan	39	1 (2.56)	2.56 (0.35–16.44)	5.6^i^
Sudan	360	12 (3.33)	3.33 (1.90–5.78)	2.3
Syria	7100	4 (0.06)	0.06 (0.02–0.15)	N/A
Uganda	1	0	0.0	6.4
Other AFR^c^	8	0	0.0	
Other EMR^d^	9	0	0.0	
Other EUR^e^	5	0	0.0	
Other WPR^f^	4	0	0.0	
Total	9623	23 (0.24)	0.24 (0.15–0.36)	

^a^Syphilis yield was calculated on adults aged 15 years and older, for ethical reasons and for ease of comparison to reference ranges from WHO

^b^No confidence intervals provided for WHO data

^c^Other AFR included Burundi, Congo, Rwanda, Cameroon, Nigeria

^d^Other EMR included Jordan, Lebanon, Djibouti, Yemen, Pakistan

^e^Other EUR included UK, St Helena, Switzerland, Turkey

^f^Other WPR included Solomon Islands, China, Taiwan or applicants with no nationality specified

^g^2011 data

^h^2010 data

^i^2013 data

*CI* confidence interval

In the multivariable analysis, those who remained at significantly higher odds for syphilis included those from AFR (aOR 4.24, 95% CI 1.21–24.82, *p* = 0.024), 35 years of age and older (35–49 years: 11.97, 1.45–99.22, *p* = 0.021; 50+ years: 12.15, 1.38–106.65, *p* = 0.024), HIV positive (10.27, 1.30–81.40, *p* = 0.027) and with a history of torture (3.27, 1.07–9.95, *p* = 0.037). Those examined in 2015–2016 remained at significantly lower odds for syphilis (2015: 0.15, 0.03–0.86, *p* = 0.033; 2016: 0.26, 0.09–0.72, *p* = 0.009) (Additional file 2: Table S3).

### Hepatitis B

Of 188 cases of hepatitis B, 130 (69%) were male and 132 (70%) were aged between 25 and 49 years. The overall testing yield for hepatitis B was 2.04% (1.77%–2.35%) and ranged by nationality from 0.58% (0.19%–1.79%) for Iraq to 12.50% (5.24%–26.96%) for South Sudan. Testing yields from Somali, Sudanese and Syrian nationals were lower than the available WHO estimates (Table 5).

**Table 5:** Hepatitis B yield (%) in tested applicants compared to estimated prevalence of chronic HBV infection (reference years 1965–2013), by country of nationality

Country of nationality	Number screened (n)	Number of cases detected	Yield in tested cohort, % (95% CI)^a^	Estimated prevalence of chronic HBV infection (HBsAg seroprevalence), 1965–2013, % (95% CI) [21]
Afghanistan	57	1	1.75 (0.24–11.61)	1.62 (1.29–2.03)
Democratic Republic of Congo	499	29	5.81 (4.07–8.24)	5.99 (5.68–6.31)
Eritrea	54	0	0.0	2.49 (2.32–2.67)
Ethiopia	251	12	4.78 (2.73–8.24)	6.03 (5.77–6.31)
Iran	14	0	0.0	0.96 (0.95–0.96)
Iraq	514	3	0.58 (0.19–1.79)	0.67 (0.65–0.70)
Palestine	28	0	0.0	1.80 (1.07–3.02)
Somalia	384	13	3.39 (1.97–5.75)	14.77 (13.77–15.84)
South Sudan	40	5	12.50 (5.24–26.96)	22.38 (20.10–24.84)
Sudan	361	21	5.82 (3.82–8.76)	9.76 (9.03–10.54)
Syria	6996	102	1.46 (1.20–1.77)	2.62 (2.17–3.17)
Uganda	2	0	0.0	9.19 (8.65–9.77)
Other AFR^b^	8	1	12.50 (1.50–57.31)	8.83 (8.82–8.83)
Other EMR^c^	9	0	0.0	3.01 (3.01–3.01)
Other EUR^d^	5	1	20.00 (2.11–74.35)	2.06 (2.06–2.06)
Other WPR^e^	3	0		5.26 (5.26–5.26)
Total	9228	188	2.04 (1.77–2.35)	

^a^Yield was calculated on adults aged 15 years and older, for ethical reasons and consistency

^b^Other AFR included Burundi, Congo, Rwanda, Cameroon, Nigeria

^c^Other EMR included Jordan, Lebanon, Djibouti, Yemen, Pakistan

^d^Other EUR included UK, St Helena, Switzerland, Turkey

^e^Other WPR included Solomon Islands, China, Taiwan or applicants with no nationality specified

*CI* confidence interval

Those who remained at significantly higher odds for hepatitis B in the multivariable analysis included men (aOR 2.66, 95% CI 1.92–3.69, *p* < 0.001), aged 25 years and older (25–34 years: 2.83, 1.69–4.77, *p* < 0.001; 35–49 years: 3.86, 2.32–6.41, *p* < 0.001; 50+: 4.07, 2.34–7.09, *p* < 0.001), from AFR (4.37, 2.91–6.55, *p* < 0.001), with a history of STI (7.65, 2.33–25.18, *p* = 0.001) and blood transfusion (2.23, 1.05–4.76, *p* = 0.038) (Additional file 2: Table S4).

### Hepatitis C

Of 38 cases of hepatitis C, 19 (50%) were male and 17 (45%) were aged 50 years and over. The overall testing yield for hepatitis C was 0.41% (0.30%–0.57%) and ranged by nationality from 0.26% (0.04–1.84%) for Somalia to 7.14% (0.92–38.84%) for Iran (Table 6).

**Table 6:** Hepatitis C yield in tested applicants compared to country prevalence estimates, where available, by country of nationality

Country of nationality	Number screened (n)	Number of cases detected (%)	Yield in tested cohort, % (95% CI)	Estimated prevalence, % (95% CI) [19, 20]^a^
Afghanistan	57	1 (1.75)	1.75 (0.24–11.61)	1.1 (0.40–1.92)
Democratic Republic of Congo	499	4 (0.80)	0.80 (0.30–2.12)	4.3 (3.2–13.7)^b^
Eritrea	54	0	0.0	N/A
Ethiopia	250	1 (0.40)	0.40 (0.06–2.79)	0.96 (0.60–1.20)
Iran	14	1 (7.14)	7.14 (0.92–38.84)	0.5 (0.20–1.00)
Iraq	517	0	0.0	0.40 (0.30–0.50)
Palestine	28	0	0.0	N/A
Somalia	382	1 (0.26)	0.26 (0.04–1.84)	N/A
South Sudan	40	0	0.0	N/A
Sudan	361	1 (0.28)	0.28 (0.04–1.94)	N/A
Syria	6994	29 (0.41)	0.41 (0.29–0.60)	2.80 (0.60–)
Uganda	2	0	0.0	N/A
Other AFR^c^	8	0	0.0	
Other EMR^d^	9	0	0.0	
Other EUR^e^	5	0	0.0	
Other WPR^f^	3	0	0.0	
Total	9223	38 (0.41)	0.41 (0.30–0.57)	

WHO regional estimates: AFR: 1.0% (0.7–1.6%); EMR: 2.3% (1.9–2.4%); EUR: 1.5% (1.2–1.5%); WPR: 0.7% (0.6–0.8%) [40]

^a^Data from Polaris Observatory HCV Collaborators, 2017 [20] unless otherwise stated. Yield was calculated on adults aged 15 years and older, for ethical reasons and consistency

^b^Data source from Gower et al. 2014 [19]

^c^Other AFR included Burundi, Congo, Rwanda, Cameroon, Nigeria

^d^Other EMR included Jordan, Lebanon, Djibouti, Yemen, Pakistan

^e^Other EUR included UK, St Helena, Switzerland, Turkey

^f^Other WPR included Solomon Islands, China, Taiwan or applicants with no nationality specified

*CI* confidence interval

Table 3 presents details of the univariable and multivariable regression analysis for hepatitis C. In the multivariable analysis, applicants aged 50 and older (6.71, 2.67–16.87, *p* < 0.001) with a history of blood transfusion (5.19, 1.70–15.88, *p* = 0.004) remained at significantly higher odds for hepatitis C infection (Additional file 2: Table S5).

## Discussion

This is the first study which reports on, and compares findings of, medical HAs for infectious diseases among a UK-bound refugee population. We found higher diagnostic yields than expected for a number of diseases, including hepatitis B.

For TB, testing yields broadly mirror WHO-estimated prevalence figures [24]. The UK programme is particularly focussed on resettlement of vulnerable refugees and, whilst the possibility of testing bias cannot be ruled out (see below), it is likely this refugee population significantly differs from the general population of the respective country. In addition, the limitations of WHO prevalence estimates have been well recognised [25] even in politically stable countries, and these limitations may be increased by political unrest present in many of the sender countries [26].

TB testing results among refugees have been highly variable. Active TB yields for German-bound Syrian asylum seekers range between 93 and 153 per 100,000 [27, 28], with some authors estimating significantly higher estimates [29]. However, other countries found significantly lower yields, as illustrated by the Dutch (22 per 100,000) [30]. Where reported in comparable pre-entry testing programmes, refugees tend to have higher TB testing yield than other migrants [5].

In our analysis of confirmed cases of TB, we demonstrated an association between active TB disease and history of TB. This is not unexpected and could reflect recurrence or reinfection and the larger proportion of cases in this cohort that came from high TB burden countries, who may have been previously exposed to TB or not completed treatment. Based on the analysis of suspected cases (Additional file 1: Appendix VI), there is additional evidence that a number of other factors may be associated with TB, including increasing age and the presence of previous household contact with TB cases. Whilst these are largely expected findings [31], they are important to help inform testing policy and guide clinical practice on the ground.

As with TB, HIV prevalence rates generally mirrored WHO figures, although we found that overall yields were higher than those that would be predicted by WHO figures. This may reflect a more vulnerable, high-risk population than expected based on the resettlement criteria of the UK programme. Among refugees from DRC, for example, HIV testing yield was five times higher than the WHO prevalence estimate, at 3.6% (2.3–5.6%) compared to the WHO estimate of 0.7% (0.6–0.9%). Overall, however, the prevalence of HIV was still relatively low (0.3%) but varied significantly between countries. Sub-Saharan African countries accounted for the majority of cases of HIV infection in this cohort, reflecting the higher prevalence rates of HIV in this region. On the other hand, the generally low prevalence seen among refugees from the Eastern Mediterranean region compared to those from sub-Saharan Africa suggests a potential practical advantage of applying a risk algorithm for determining which individuals should be tested.

Our analysis identified a number of factors, both demographic and behavioural, which increased the odds of HIV infection, including being female between the ages of 35–49, of sub-Saharan African nationality and with a history of STI. The increased vulnerability of women to HIV infection stemming from biological, social, behavioural and structural risk factors is well recognised [32, 33]. The finding of syphilis being associated with a reported history of torture is interesting, however perhaps not surprising given that more than half (12/23; 5 female, 7 male) of syphilis cases are from Sudan, where experiences of torture were generally more prevalent (reported in 21.43% of Sudanese applicants). The higher odds of syphilis among individuals with HIV are not unexpected and again reflect the biological mechanisms and similar risk factors which facilitate transmission.

A large number of refugees were identified to have hepatitis B infection in this cohort. Overall prevalence was over 2% but, as with other infections, we found that prevalence varied substantially between countries and with respect to WHO estimates. Whilst sub-Saharan African countries had particularly high prevalence, Syrian refugees accounted for most cases. In particular, testing yields for refugees from Somalia (3.39%, 1.97–5.75%), Sudan (5.82%, 3.82–8.76%) and South Sudan (12.50%, 5.24–26.96%) were noticeably lower compared to prevalence estimates (Table 5), which could reflect the limitations of prevalence estimates from these countries, but also that the refugee population may be different to the general population. An additional consideration is that these countries may have WHO-recommended universal and selective hepatitis B vaccination programmes, although vaccine coverage is unlikely to be optimal in countries with fragile infrastructures and during conflicts. The disparity between observed testing yield and country prevalence estimates therefore needs to be explored further. The high yield from the other European region category (20.00%, 2.11–74.35) reflects higher rates associated with refugees from Turkey [34, 35], an intermediate endemicity country, but the low numbers are noted [36].

We demonstrated that a number of factors are associated with increased odds of hepatitis B infection, including being male, increasing age, sub-Saharan African nationality and a history of STI and blood transfusion. It is likely that male predominance may be due to adult exposures more associated with males, and should be explored further.

Hepatitis C testing yield was considerably lower (0.41%) than seen for hepatitis B, although again this varied by nationality and in comparison with prevalence estimates, likely reflecting different risk exposure. We demonstrated that the main factors associated with increased odds of hepatitis C were older age (> 50 years) and history of blood transfusion, yet unlike hepatitis B, there was no association with geographic region. The strong association with blood transfusion particularly among the older age groups likely reflects the lack of routine blood-borne virus testing in many low- and middle-income countries, possible iatrogenic transmission through reusing of needles and medical equipment, and potentially chronic infection in some cases. It is interesting, although not totally surprising, that illicit drug use was not reported, considering the high prevalence of this exposure in some countries; however, this is not necessarily a dominant risk factor in those countries from which the majority of screened refugees originate.

The current HA programme run by IOM undertakes a significant number of tests in this vulnerable refugee population. Our novel work highlights that prevalence of infectious diseases varies widely, raising the possibility of changing testing from a blanket modality to a more nuanced, risk-based model that targets those at highest risk. Our findings also demonstrate that refugees are not a homogeneous group and provides a baseline for further evaluation of the effectiveness of the HA in facilitating initial linkages with primary care and in the years following resettlement.

Given that the primary aim of the UK programme and HA is to facilitate early integration and linkage of the refugee to appropriate health and social services in the UK, it is important that the HA is tailored with this end goal in mind and conducted according to what is appropriate for the individual, performed with the voluntarily provided informed consent of the individual. Informed consent is a key element in the protocol [14], yet it is important to be conscious that resettlement circumstances may potentially affect the freedom of consent.

Our study benefits from a large, well completed and comprehensive dataset of UK-bound refugee testing. Nevertheless, these observational data have a number of limitations, including data recording issues with potential for incomplete data or misclassification. Whilst the dataset was not subject to the routine IOM validation process, there has been rigorous data cleaning and validation before analysis to minimise such issues and it is therefore likely that the potential for misclassification is small and occurring at random. For active TB, IOM provided a rigorous case ascertainment exercise which verified status according to culture confirmation with the attending physicians in the field for all suspected cases.

Detecting diseases depends on the availability and quality of testing sites and it is possible that this may lead to testing bias. Most diseases require confirmatory tests and we employed very robust algorithms for case definition, so any testing bias would likely lead to case under-ascertainment. We have analysed the impact of assessment site on disease prevalence and think that these effects are likely minimal. For active TB, there is a possibility of confirmed case under-ascertainment owing to the methodology used.

Detected disease yields in our study are often similar, but sometimes different compared with WHO-estimated disease prevalence. It is expected that infectious disease risk and prevalence in this refugee population is different from the general population, not least because of differences in socioeconomic circumstances, access to care and/or accommodation, including overcrowding and camp conditions, which would minimise the generalisability of our yield as disease prevalence for the specific countries. Likewise, our study population is generalisable to UK-bound refugee populations and likely to refugee populations to other destination countries with similar programmes (e.g. USA, Canada, Australia, New Zealand). However, the generalisability of our results to other migrant or asylum seeker populations is limited due to differences in epidemiological profiles, socioeconomic status and possible selection bias (e.g. due to different selection criteria of resettlement programmes). Nevertheless, our findings provide an important snapshot into infectious disease risk of UK-bound refugees and yields important lessons to inform public health measures in this vulnerable population.

The limitations in self-reporting of risk factors, particularly if potentially considered criminal or stigmatising in the country of origin, should also be considered here. Whilst the null report of illicit drug use among hepatitis C cases may be real, it could also reflect lack of disclosure in response to fear of stigma or legal implications affecting rights to resettlement.

A further limitation is that the data recorded provided disease prevalence on a select group of refugees, predominantly from Africa and the Middle East, with fewer from Asia, who may have had a different infectious disease profile.

## Conclusions

Our paper compares the findings of systematic infectious disease testing within the UK refugee programme with WHO prevalence estimates and comparable testing programmes, and elicits factors associated with case positivity. Whilst the magnitude of infectious disease findings was unexpected for some diseases in some settings, most of our study corroborates findings from similar programmes [5, 7, 31]. There are a number of important lessons, most notably the geographical variation of testing yields, which may help design a more targeted approach to testing. It is worth noting, for example, that HIV and syphilis rates tend to be very low in the Eastern Mediterranean Region, as expected on the basis of WHO rates, and this fact could help inform testing policies, which currently do not take country-level disease prevalence into consideration in their advice. The most recent iteration of the UK HA protocol [14] has made progress in this regard, tailoring testing policies for hepatitis B and C on the basis of personal risk factors and prevalence in the area of origin, which has also been suggested in the literature [37]. These findings provide evidence to potentially support a similar approach for other infectious diseases in some settings. Further evaluation of the utility of the HA in linking refugees with primary care and social services on resettlement in the UK would also be a valuable next step in informing policy. Our paper is the first exploration of such issues and further, more detailed analysis is needed to guide best practice in refugee health and infectious disease testing in particular.

## Additional files

Additional file 1: Supplementary material. (DOCX 69 kb)

Additional file 2: Supporting tables: logistic regression analysis. **Table S1.** Testing yield and logistic regression analysis of the TB test cohort^†^ (outcome = active TB). **Table S2.** Testing yield and logistic regression analysis of the HIV test cohort^†^ (outcome = HIV positive). **Table S3.** Testing yield and logistic regression analysis of the syphilis test cohort^†^ (outcome = syphilis positive). **Table S4.** Testing yield and logistic regression analysis of the hepatitis B test cohort^†^ (outcome= hepatitis B positive). **Table S5.** Testing yield and logistic regression analysis of the hepatitis C test cohort^†^ (outcome: hepatitis C positive). (DOCX 62 kb)

## Abbreviations

AFR: WHO African Region
aOR: adjusted odds ratio
CI: confidence interval
DRC: Democratic Republic of Congo
EU: European Union
HA: health assessment
HIV: Human immunodeficiency virus
IOM: International Organization for Migration
OR: odds ratio
STI: sexually transmitted infection
TB: Tuberculosis
UNHCR: United Nations High Commission for Refugees
VCRS: Vulnerable Children Resettlement Scheme
VPRS: Vulnerable Persons Resettlement Programme
WHO: World Health Organization

## Footnotes

1 Close family members or, in some cases, history of time spent in the UK.

2 Google searches in the UK for ‘refugees’ increased nearly 100-fold between March and September 2015 and peaked in September 2015 over a 5-year period, Additional file 1: Appendix III).

3 All cases, under all UK resettlement programmes, are subject to health assessments according to UK protocol [41].

4 Protocol jointly developed by the UK Home Office, Public Health England and IOM, and updated on an ad hoc basis, as appropriate. Most recently updated in July 2017. The data analysed in this study were collected according to pre-2015 versions of the protocol.

5 Note that, whilst refugees with the same case number belong to the same immediate family, refugees with different case numbers may still be related. That is, they may be members of the same extended family (IOM, personal communication). It was not possible to account for correlation that may occur between individuals of the same extended family in the cluster analysis.

6 WHO EMR: Afghanistan, Iran, Iraq, Jordan, Lebanon, Somalia, Sudan, Syria, Djibouti, Yemen, Pakistan.

7 WHO AFR: Burundi, Congo, Democratic Republic of Congo, Eritrea, Ethiopia, Rwanda, South Sudan, Uganda, Cameroon, Nigeria.

8 UNHCR conducts a Refugee Status Determination interview individually with each asylum seeker. According to the UNHCR Procedural Standards for Refugee Status Determination under UNHCR's mandate (2005), each accompanying adult family member/dependent should have an individual and confidential Registration Interview [42].
