# Supplementary material for: Infectious disease testing of UK-bound refugees: a population-based, cross-sectional study
Source: BMC Med. 2018 Aug 28;16:143. doi: 10.1186/s12916-018-1125-4 (PMC6112114; doi:10.1186/s12916-018-1125-4)
Supplement: Supplementary file 1 — Supplementary material. (DOCX 69 kb) [file 12916_2018_1125_MOESM1_ESM.docx]

**Additional file 1**

**Appendix I**

Disease case definitions

Disease case definitions were pre-defined according to criteria specified in the UK HA protocol and UK technical instructions and recorded in the form by the IOM panel physician at the time of the HA under a case-specific variable.

For the purpose of this study, testing cohorts were defined as all individuals to have undergone minimum testing requirements (as per the HA protocol)^[[1]](#footnote-2)^ for a specific disease. Cases were then identified from within each testing cohort and verified in the data cleaning stage against the protocol algorithms, and the laboratory and radiology reports and physician’s notes (Table 7).

Table 7. Criteria used to define testing cohorts and define and reclassify disease cases

| **Disease** | **Testing** **cohort** | **Case definition** | **Reclassification details** |
| --- | --- | --- | --- |
| Active TB | Anyone to attend TB screening. | Any case recorded as culture-positive. | N/A |
| HIV | Anyone to undergo a primary HIV test. | IOM-identified* HIV case with a positive primary test and positive confirmatory test as per national diagnostic guidelines; or IOM-identified case with three positive test results (to account for different availability of testing methods). | One case identified as HIV-positive by IOM was recoded as HIV-negative on the basis that it did not meet the algorithm criteria (only two rapid diagnostic tests were performed) and physician’s notes did not support the diagnosis. Two algorithmically HIV-negative cases were recoded as HIV-positive on the basis of the physician’s notes, which indicated prior HIV-positive diagnosis and ongoing prophylactic treatment. |
| Syphilis | Anyone to undergo an initial non-treponemal test, as well as a confirmatory treponemal test when the initial test result was positive or indeterminate. | Positive initial non-treponemal test followed by a positive confirmatory treponemal test, as per national guidelines. | N/A |
| Hep B | Anyone to undergo at least a primary hepatitis B test. | Positive HBsAg test and a positive confirmatory test, as per national guidelines. | N/A |
| Hep C | Anyone to undergo at least a primary hepatitis C test. | Positive anti-HCV test and a positive confirmatory test, as per national guidelines. | N/A |

In addition to following the specific criteria for defining cases outlined in the case definitions, all cases were also corroborated against the physician’s notes and/or laboratory notes to ensure rigour.

Testing algorithms followed by IOM

*Cases are defined by IOM according to the following algorithms:

HIV:

- The primary first line assay (A1) to be used is a fourth generation assay, such as the ELISA test or equivalent (EIA), which tests for HIV antibody and p24 antigen simultaneously. If it is negative, no further testing is required and the sample is reported as negative. If positive or indeterminate, a secondary test (A2) and a confirmatory test (A3) should be performed, on the same sample.
- The secondary test to be used is ELISA (different brand/ different manufacturer) or equivalent (EIA).
- The confirmatory test (third test; A3) to be used is Western-blot (WB), where and when available; or another ELISA test of different manufacturer, or a rapid test for HIV 1 and HIV 2 when WB testing is not available.
- In case of non-availability of ELISA and WB, a set of three different WHO approved rapid tests for both HIV 1 and HIV 2 can be used, in accordance with the country of testing regulations and accepted HIV testing protocol.
- Equivocal or indeterminate results are to be re-tested after two weeks.
- Confirmation of an HIV diagnosis should prompt the physician to consider testing for other STIs, such as chlamydia or syphilis.

Syphilis:

- The testing algorithm includes the primary non-specific testing test (Rapid Plasma Reagin [RPR], or Venereal Disease Research Laboratory [VDRL] test).
- When the initial testing test is negative, the test result is reported as negative.
- Positive testing tests should be confirmed with a different treponemal (specific) test, such as the TP Haemagglutination Assay, the Abbott Determine TP test (rapid test) or equivalent.
- When the confirmatory specific test is negative, the test result is reported as negative.
- When the confirmatory specific test is positive, the test result is reported as positive.

Hepatitis B & C:

- HBV testing should start with HBV surface antigen (HBsAg) with further HBV markers if HBsAg positive. HCV testing should start with antibodies to HCV (anti-HCV), followed by HCV RNA if anti-HCV positive
- “Further HBV markers” refer to additional markers used to confirm and/or classify the HBV diagnosis, for example, by level of infectivity. These are performed in line with the protocol however some IOM clinics slightly vary in the additional tests used, for example Middle East IOM clinics use Hep B e-antigen (HBeAg) and anti-HB-core antigen (anti-HBcAg) as supplementary markers following a positive HBsAg test, whereas IOM Burundi uses HBV viral DNA.

TB:

- Active TB cases were identified through a two-step process. Suspected TB cases were initially identified based on a database-based definition comprising clinical signs and symptoms, radiological findings and laboratory results. These were then individually verified by each IOM clinic and confirmed cases of active TB were defined based on positive culture. See the UK TB technical instructions^[[2]](#footnote-3)^ for complete details of TB testing process.

Other definitions

Nationality: based on country of birth or passport.

Data management

Duplicates (n=935) were excluded in the data cleaning stage. Where applicants had more than one HA record only the completed HA or most recent record was retained for analysis. Applicants whose health assessment was not completed (n=686) or with no nationality specified (n=3) were excluded from the sociodemographic summary of the cohort. Additional exclusion criteria were applied prior to analysis (Figure 1).

Countries of examination

Health assessments took place in IOM clinics in 14 countries: Burundi, Egypt, Greece, Iraq, Jordan, Kenya, Lebanon, Namibia, Rwanda, Syrian Arab Republic, Turkey, Uganda, United Arab Emirates.

Countries of nationality

Nationals of 28 countries were represented: Afghanistan, Burundi, Congo, Democratic Republic of Congo, Eritrea, Ethiopia, Iran, Iraq, Jordan, Lebanon, Rwanda, St Helena, Solomon Islands, Somalia, South Sudan, Sudan, Switzerland, Syrian Arab Republic, Turkey, Uganda, UK, Cameroon, China, Djibouti, Nigeria, Palestine, Taiwan, Yemen, Pakistan.

**Appendix II**

Table 8. Variables adjusted for in multivariable logistic regression analysis, by outcome. Each outcome represents a single model.

|  | **Outcome** | | | | |
| --- | --- | --- | --- | --- | --- |
| **Variable** | **TB** | **HIV** | **Syphilis** | **Hepatitis B** | **Hepatitis C** |
| Age | X | X | X | X | X |
| Sex | X | X | X | X | X |
| WHO region of nationality | X | X | X | X | X |
| Examination year | X | X | X | X | X |
| History of displacement | X | X | X | X | X |
| History of blood transfusion |  | X | X | X | X |
| History of torture |  | X | X | X | X |
| History of alcohol intake |  | X | X | X | X |
| History of illicit drug use |  | X | X | X | X |
| History of tattoos |  | X | X | X | X |
| History of STI |  | X |  | X | X |
| Current HIV infection |  |  | X |  |  |
| History of TB | X |  |  |  |  |
| History of household member with TB |  |  |  |  |  |
| Current pregnancy |  |  |  |  |  |
| Case number (cluster analysis for families) | X | X | X | X | X |

X, variable included in multivariable logistic regression model for the selected outcome.

**Appendix III**

Google searches in the UK for “refugees” increased nearly 100-fold between March and September 2015 and peaked in September 2015 over a 5 year period.

Figure 1. UK search term (‘refugees’) interest over a 5 year period (2012-2017) according to Google Trends.

Search term (‘refugees’) interest over time (2012-2017) according to Google Trends. Numbers represent search interest relative to the highest point on the chart for the given region and time. A value of 100 is the peak popularity for the term. A value of 50 means that the term is half as popular. Likewise a score of 0 means the term was less than 1% as popular as the peak. Source: <https://trends.google.co.uk/trends/explore?date=today%205-y&geo=GB&q=syrian%20refugees> Download date: 29 July 2016.

**Appendix IV**

Table 9. Sociodemographic summary of individuals included and excluded from the analysis (those aged >15 years versus those <15 years of age).

|  | Included in analysis  (n=9759) | | Excluded from analysis  (n=7970) | |
| --- | --- | --- | --- | --- |
| ***Median age, years*** |  | |  | |
| Mean | 33.5 | | 6.46 | |
| Median | 31.8 | | 6.07 | |
| Range | (15.0-86.9) | | (<1mo-14.9) | |
| ***Sex*** | **N** | **%** | **N** | **%** |
| Male  Female | 4743 5016 | 48.6 51.4 | 3641  4329 | 45.68  54.32 |
| ***Nationality*** | **N** | **%** | **N** | **%** |
| Afghanistan | 63 | 0.65 | 47 | 0.59 |
| DRC | 570 | 5.84 | 399 | 5.01 |
| Eritrea | 59 | 0.6 | 22 | 0.28 |
| Ethiopia | 290 | 2.97 | 183 | 2.3 |
| Iran | 15 | 0.15 | 6 | 0.08 |
| Iraq | 540 | 5.53 | 361 | 4.53 |
| Somalia | 562 | 5.76 | 460 | 5.77 |
| South Sudan | 40 | 0.41 | 27 | 0.34 |
| Sudan | 369 | 3.78 | 317 | 3.98 |
| Syria | 7195 | 73.73 | 6101 | 76.55 |
| Palestine | 28 | 0.29 | 17 | 0.21 |
| Uganda | 2 | 0.02 | 0 | 0 |
| Other AFRO | 8 | 0.08 | 8 | 0.1 |
| Other EMRO | 9 | 0.09 | 10 | 0.13 |
| Other EURO | 5 | 0.05 | 11 | 0.14 |
| Other WPRO | 4 | 0.04 | 1 | 0.01 |
| *WHO region* | **N** | **%** | **N** | **%** |
| EMRO  AFRO  EURO  WPRO | 8753  969  33  4 | 89.69  9.93  0.34  0.04 | 7302  639  28  1 | 91.62  8.02  0.35  0.01 |

Note that additional selection criteria were applied prior to analysis and are described in Figure 1.

**Appendix V**

STROBE Statement—Checklist of items that should be included in reports of ***cross-sectional studies***

|  | **Item No** | **Recommendation** | **Done (Y/N)** |
| --- | --- | --- | --- |
| **Title and abstract** | 1 | (*a*) Indicate the study’s design with a commonly used term in the title or the abstract | **Y** |
|  |  | (*b*) Provide in the abstract an informative and balanced summary of what was done and what was found | **Y** |
| **Introduction** | | |  |
| Background/rationale | 2 | Explain the scientific background and rationale for the investigation being reported | **Y** |
| Objectives | 3 | State specific objectives, including any prespecified hypotheses | **Y** |
| **Methods** | | |  |
| Study design | 4 | Present key elements of study design early in the paper | **Y** |
| Setting | 5 | Describe the setting, locations, and relevant dates, including periods of recruitment, exposure, follow-up, and data collection | **Y** |
| Participants | 6 | (*a*) Give the eligibility criteria, and the sources and methods of selection of participants | **Y** |
| Variables | 7 | Clearly define all outcomes, exposures, predictors, potential confounders, and effect modifiers. Give diagnostic criteria, if applicable | **Y** |
| Data sources/ measurement | 8* | For each variable of interest, give sources of data and details of methods of assessment (measurement). Describe comparability of assessment methods if there is more than one group | **Y** |
| Bias | 9 | Describe any efforts to address potential sources of bias | **Y** |
| Study size | 10 | Explain how the study size was arrived at | **Y** |
| Quantitative variables | 11 | Explain how quantitative variables were handled in the analyses. If applicable, describe which groupings were chosen and why | **Y** |
| Statistical methods | 12 | (*a*) Describe all statistical methods, including those used to control for confounding | **Y** |
|  |  | (*b*) Describe any methods used to examine subgroups and interactions | **Y** |
|  |  | (*c*) Explain how missing data were addressed | **Y** |
|  |  | (*d*) If applicable, describe analytical methods taking account of sampling strategy | **N/A** |
|  |  | (*e*) Describe any sensitivity analyses | **N/A** |
| **Results** | | |  |
| Participants | 13* | (a) Report numbers of individuals at each stage of study—eg numbers potentially eligible, examined for eligibility, confirmed eligible, included in the study, completing follow-up, and analysed | **Y** |
|  |  | (b) Give reasons for non-participation at each stage | **Y** |
|  |  | (c) Consider use of a flow diagram | **Y** |
| Descriptive data | 14* | (a) Give characteristics of study participants (eg demographic, clinical, social) and information on exposures and potential confounders | **Y** |
|  |  | (b) Indicate number of participants with missing data for each variable of interest | **Y** |
| Outcome data | 15* | Report numbers of outcome events or summary measures | **Y** |
| Main results | 16 | (*a*) Give unadjusted estimates and, if applicable, confounder-adjusted estimates and their precision (eg, 95% confidence interval). Make clear which confounders were adjusted for and why they were included | **Y** |
|  |  | (*b*) Report category boundaries when continuous variables were categorized | **Y** |
|  |  | (*c*) If relevant, consider translating estimates of relative risk into absolute risk for a meaningful time period |  |
| Other analyses | 17 | Report other analyses done—eg analyses of subgroups and interactions, and sensitivity analyses | **Y** |
| **Discussion** | | |  |
| Key results | 18 | Summarise key results with reference to study objectives | **Y** |
| Limitations | 19 | Discuss limitations of the study, taking into account sources of potential bias or imprecision. Discuss both direction and magnitude of any potential bias | **Y** |
| Interpretation | 20 | Give a cautious overall interpretation of results considering objectives, limitations, multiplicity of analyses, results from similar studies, and other relevant evidence | **Y** |
| Generalisability | 21 | Discuss the generalisability (external validity) of the study results |  |
| **Other information** | | |  |
| Funding | 22 | Give the source of funding and the role of the funders for the present study and, if applicable, for the original study on which the present article is based | **Y** |

*Give information separately for exposed and unexposed groups.

**Note:** An Explanation and Elaboration article discusses each checklist item and gives methodological background and published examples of transparent reporting. The STROBE checklist is best used in conjunction with this article (freely available on the Web sites of PLoS Medicine at http://www.plosmedicine.org/, Annals of Internal Medicine at http://www.annals.org/, and Epidemiology at http://www.epidem.com/). Information on the STROBE Initiative is available at www.strobe-statement.org.

**Appendix VI**

Table 10. Sensitivity analysis of suspected versus confirmed active TB logistic regression models.

|  | Suspected TB cases (n=134) | | | | Confirmed active TB cases (n=9) | | | | |
| --- | --- | --- | --- | --- | --- | --- | --- | --- | --- |
| Variable | Unadjusted OR (95% CI) | P value | Adjusted OR (95% CI) | P value | Unadjusted OR (95% CI) | P value | Adjusted OR (95% CI) | P value | |
| **ACTIVE TB** |  |  |  |  |  |  |  | |  |
| Age group (years) |  |  |  |  |  |  |  | |  |
| 15-24* |  |  |  |  |  |  |  | |  |
| 25-34 | **3.07 (1.47-6.41)** | 0.003 | **3.21 (1.52-6.78)** | 0.002 | 1.61(0.30-8.81) | 0.581 | 1.28 (0.22-7.30) | | 0.784 |
| 35-49 | **5.42(2.65-11.07)** | <0.001 | **5.44(2.64-11.21)** | <0.001 | 1.53(0.26-9.18) | 0.640 | 1.24(0.20-7.82) | | 0.818 |
| 50+ | **11.82(5.74-24.33)** | <0.001 | **12.53(6.17-25.47)** | <0.001 | No obs |  | No obs | |  |
| Sex |  |  |  |  |  |  |  | |  |
| Female* |  |  |  |  |  |  |  | |  |
| Male | 1.39(0.99-1.96) | 0.060 | 1.37(0.97-1.93) | 0.074 | 2.12(0.53-8.47) | 0.289 | 1.67 (0.33-8.46) | | 0.535 |
| WHO region of nationality |  |  |  |  |  |  |  | |  |
| AFR | **1.79(1.13-2.84)** | 0.013 | 1.38(0.74-2.56) | 0.309 | **7.25(1.94-27.05)** | **0.003** | 4.83 (0.97-24.01) | | 0.054 |
| EUR | No obs |  | No obs |  | No obs |  | No obs | |  |
| EMR* |  |  |  |  |  |  |  | |  |
| WPR | No obs |  | No obs |  | No obs |  | No obs | |  |
| WHO region of examination |  |  |  |  |  |  |  | |  |
| AFR | **3.09(2.14-4.45)** | <0.001 |  |  | **13.67(2.65-70.49)** | 0.002 |  | |  |
| EUR | 0.42(0.17-1.03) | 0.058 |  |  | 6.97(0.98-49.51) | 0.052 |  | |  |
| EMR* |  |  |  |  |  |  |  | |  |
| Year of examination |  |  |  |  |  |  |  | |  |
| 2013 | No obs |  | No obs |  | No obs |  | No obs | |  |
| 2014 | **3.15(1.84-5.41)** | <0.001 | **2.45(1.26-4.80)** | 0.009 | 2.20(0.23-21.16) | 0.496 | 1.06 (0.04-28.56) | | 0.972 |
| 2015 | 0.72(0.40-1.28) | 0.259 | 0.62(0.30-1.27) | 0.190 | No obs |  | No obs | |  |
| 2016 | 0.89(0.59-1.33) | 0.560 | 0.84(0.56-1.27) | 0.415 | 1.18(0.28-4.94) | 0.822 | 1.18 (0.20-6.84) | | 0.854 |
| 2017* |  |  |  |  |  |  |  | |  |
| History of household member with TB |  |  |  |  |  |  |  | |  |
| No* |  |  |  |  |  |  |  | |  |
| Yes | **10.45(4.37-24.98)** | <0.001 | **7.10(3.05-16.52)** | <0.001 | No obs |  |  | |  |
| HIV positive |  |  |  |  |  |  |  | |  |
| No* |  |  |  |  |  |  |  | |  |
| Yes | **4.37(1.04-18.41)** | 0.044 | 1.74(0.30-10.08) | 0.539 | No obs |  |  | |  |
| History of displacement |  |  |  |  |  |  |  | |  |
| No* |  |  |  |  |  |  |  | |  |
| Yes | 0.72(0.50-1.04) | 0.081 | 0.72(0.47-1.10) | 0.130 | 0.66(0.17-2.64) | 0.558 | 0.88 (0.22-3.46) | | 0.853 |
| Contact with TB patients |  |  |  |  |  |  |  | |  |
| No* |  |  |  |  |  |  |  | |  |
| Yes | **13.43(4.56-39.52)** | <0.001 |  |  | **48.62(5.86-403.32)** | **<0.001** |  | |  |
| History of TB |  |  |  |  |  |  |  | |  |
| No* |  |  |  |  |  |  |  | |  |
| Yes | **38.43(16.94-87.20)** | <0.001 | **23.46(8.76-62.82)** | <0.001 | **202.63(47.88-857.47)** | **<0.001** | **145.53 (25.99-814.84)** | | <0.001 |
| Currently pregnant |  |  |  |  |  |  |  | |  |
| No* |  |  |  |  |  |  |  | |  |
| Yes | 0.39(0.05-2.80) | 0.350 | 0.71(0.10-5.06) | 0.730 | No obs |  |  | |  |

1. E.g. for syphilis, this would include all those who underwent an initial non-treponemal test, as well as all those who underwent a confirmatory treponemal test when the initial test result was positive or indeterminate. [↑](#footnote-ref-2)
2. UK tuberculosis technical instructions. Published 25 September 2013. Source: https://www.gov.uk/government/publications/uk-tuberculosis-technical-instructions [↑](#footnote-ref-3)
