# Supplementary material for: Infectious disease testing of UK-bound refugees: a population-based, cross-sectional study
Source: BMC Med. 2018 Aug 28;16:143. doi: 10.1186/s12916-018-1125-4 (PMC6112114; doi:10.1186/s12916-018-1125-4)
Supplement: Supplementary file 2 — Supporting tables: logistic regression analysis. Table S1. Testing yield and logistic regression analysis of the TB test cohort† (outcome = active TB). Table S2. Testing yield and logistic regression analysis of the HIV test cohort† (outcome = HIV positive). Table S3. Testing yield and logistic regression analysis of the syphilis test cohort† (outcome = syphilis positive). Table S4. Testing yield and logistic regression analysis of the hepatitis B test cohort† (outcome= hepatitis B positive). Table S5. Testing yield and logistic regression analysis of the hepatitis C test cohort† (outcome: hepatitis C positive). (DOCX 62 kb) [file 12916_2018_1125_MOESM2_ESM.docx]

Additional file 2

**Table S1.** Testing yield and logistic regression analysis of the TB test cohort^†^ (outcome = active TB)

| Variable | Total number screened | Number of positive cases (%) | Unadjusted OR (95% CI) | P value | Adjusted OR (95% CI) | P value |
| --- | --- | --- | --- | --- | --- | --- |
|  |  |  |  |  |  |  |
| **ACTIVE TB** | ***9759*** | ***n = 9*** |  |  |  |  |
| Age group (years) |  |  |  |  |  |  |
| 15-24* | 2649 | 2 |  |  |  |  |
| 25-34 | 3286 | 4 | 1.61(0.30-8.81) | 0.581 | 1.28 (0.22-7.30) | 0.784 |
| 35-49 | 2593 | 3 | 1.53(0.26-9.18) | 0.640 | 1.24(0.20-7.82) | 0.818 |
| 50+ | 1110 | 0 | No obs |  | No obs |  |
| Sex |  |  |  |  |  |  |
| Female* | 5016 | 3 |  |  |  |  |
| Male | 4743 | 6 | 2.12(0.53-8.47) | 0.289 | 1.67 (0.33-8.46) | 0.535 |
| WHO region of nationality |  |  |  |  |  |  |
| AFR | 969 | 4 | **7.25(1.94-27.05)** | 0.003 | 4.83 (0.97-24.01) | 0.054 |
| EUR | 33 | 0 | No obs |  | No obs |  |
| EMR* | 8753 | 5 |  |  |  |  |
| WPR | 4 | 0 | No obs |  | No obs |  |
| WHO region of examination |  |  |  |  |  |  |
| AFR | 1350 | 5 | **13.67(2.65-70.49)** | 0.002 |  |  |
| EUR | 1052 | 2 | 6.97(0.98-49.51) | 0.052 |  |  |
| EMR* | 7269 | 2 |  |  |  |  |
| Year of examination | ***n = 9638*** | ***n = 9*** |  |  |  |  |
| 2013 | 3 | 0 | No obs |  | No obs |  |
| 2014 | 475 | 1 | 2.20(0.23-21.16) | 0.496 | 1.06 (0.04-28.56) | 0.972 |
| 2015 | 1615 | 0 | No obs |  | No obs |  |
| 2016 | 4419 | 5 | 1.18(0.28-4.94) | 0.822 | 1.18 (0.20-6.84) | 0.854 |
| 2017* | 3126 | 3 |  |  |  |  |
| History of household member with TB |  |  |  |  |  |  |
| No* | 9710 | 9 |  |  |  |  |
| Yes | 49 | 0 | No obs |  |  |  |
| HIV positive |  |  |  |  |  |  |
| No* | 9654 | 9 |  |  |  |  |
| Yes | 35 | 0 | No obs |  |  |  |
| History of displacement |  |  |  |  |  |  |
| No* | 2424 | 3 |  |  |  |  |
| Yes | 7335 | 6 | 0.66(0.17-2.64) | 0.558 | 0.88 (0.22-3.46) | 0.853 |
| Contact with TB patients |  |  |  |  |  |  |
| No* | 9733 | 8 |  |  |  |  |
| Yes | 26 | 1 | **48.62(5.86-403.32)** | <0.001 |  |  |
| History of TB |  |  |  |  |  |  |
| No* | 9732 | 6 |  |  |  |  |
| Yes | 27 | 3 | **202.63(47.88-857.47)** | <0.001 | **145.53 (25.99-814.84)** | <0.001 |
| Currently pregnant |  |  |  |  |  |  |
| No* | 9575 | 9 |  |  |  |  |
| Yes | 183 | 0 | No obs |  |  |  |

OR, odds ratio; CI, confidence interval. Unadjusted and adjusted odds ratios calculated using generalised estimating equations logistic regression. Contact with TB patients were removed from the final multivariable model to reduce collinearity with history of household member with TB. Examination region was removed to reduce collinearity with WHO region of nationality.
†Children under the age of 15 were excluded from the logistic regression analysis.
*Comparison group

**Table S2.** Testing yield and logistic regression analysis of the HIV test cohort^†^ (outcome = HIV positive).

| Variable | Total number screened | Number of positive cases (%) | Unadjusted OR (95% CI) | P value | Adjusted OR (95% CI) | P value |
| --- | --- | --- | --- | --- | --- | --- |
|  |  |  |  |  |  |  |
| **HIV** | ***n = 9689*** | ***n=35*** |  |  |  |  |
| Age group (years) |  |  |  |  |  |  |
| 15-24* | 2631 | 4 |  |  |  |  |
| 25-34 | 3284 | 8 | 1.60(0.48-5.33) | 0.441 | 1.19(0.36-3.89) | 0.777 |
| 35-49 | 2591 | 19 | **4.85(1.65-14.28)** | 0.004 | **5.76(2.05-16.22)** | 0.001 |
| 50+ | 1105 | 4 | 2.39(0.60-9.56) | 0.219 | 1.48(0.29-7.50) | 0.639 |
| Sex |  |  |  |  |  |  |
| Female* | 4987 | 28 |  |  |  |  |
| Male | 4702 | 7 | **0.26(0.12-0.61)** | 0.002 | **0.18(0.07-0.50)** | 0.001 |
| WHO region of nationality |  |  |  |  |  |  |
| AFR | 959 | 25 | **23.25(11.13-48.55)** | <0.001 | **51.72(20.67-129.39)** | <0.001 |
| EUR | 31 | 0 | No obs |  | No obs |  |
| EMR* | 8695 | 10 |  |  |  |  |
| WPR | 4 | 0 | No obs |  | No obs |  |
| WHO region of examination |  |  |  |  |  |  |
| AFR | 1334 | 28 | **22.41(9.77-51.40)** | <0.001 |  |  |
| EUR | 1032 | 0 | No obs |  |  |  |
| EMR* | 7323 | 7 |  |  |  |  |
| Year of examination | ***n = 9611*** | ***n = 35*** |  |  |  |  |
| 2013 | 3 | 0 | No obs |  | No obs |  |
| 2014 | 470 | 5 | 1.94(0.71-5.28) | 0.195 | **0.11(0.02-0.55)** | 0.007 |
| 2015 | 1610 | 6 | 0.68(0.27-1.73) | 0.421 | **0.28(0.08-0.97)** | 0.043 |
| 2016 | 4408 | 7 | **0.29(0.12-0.70)** | 0.006 | **0.35(0.13-0.97)** | 0.043 |
| 2017* | 3115 | 17 |  |  |  |  |
| History of surgical intervention |  |  |  |  |  |  |
| No* | 6323 | 26 |  |  |  |  |
| Yes | 3366 | 9 | 0.65(0.30-1.39) | 0.265 |  |  |
| History of STI |  |  |  |  |  |  |
| No* | 9668 | 24 |  |  |  |  |
| Yes | 21 | 11 | **442.01(171.73-1137.71)** | <0.001 | **1521.54(342.76-6754.23)** | <0.001 |
| History of tattoos |  |  |  |  |  |  |
| No* | 9147 | 34 |  |  |  |  |
| Yes | 542 | 1 | 0.50(0.07-3.63) | 0.489 | 0.03(<0.001-0.96) | 0.047 |
| History of blood transfusion |  |  |  |  |  |  |
| No* | 9477 | 31 |  |  |  |  |
| Yes | 212 | 4 | **5.86(2.05-16.75)** | 0.001 | 3.67(0.51-26.22) | 0.195 |
| History of torture |  |  |  |  |  |  |
| No* | 8853 | 28 |  |  |  |  |
| Yes | 836 | 7 | **2.66(1.16-6.11)** | 0.021 |  |  |
| History of displacement |  |  |  |  |  |  |
| No* | 2403 | 16 |  |  |  |  |
| Yes | 7286 | 19 | **0.39(0.20-0.76)** | 0.006 | 0.46(0.19-1.12) | 0.086 |
| History of alcohol intake |  |  |  |  |  |  |
| No* | 9640 | 34 |  |  |  |  |
| Yes | 49 | 1 | 5.89(0.79-43.87) | 0.084 | 1.62(0.0003-8505.01) | 0.912 |
| History of illicit drug use |  |  |  |  |  |  |
| No* | 9663 | 33 |  |  |  |  |
| Yes | 26 | 2 | **24.32(5.52-107.09)** | <0.001 | 8.88(0.04-1985.18) | 0.429 |
|  |  |  |  |  |  |  |

OR, odds ratio; CI, confidence interval. Unadjusted and adjusted odds ratios calculated using generalised estimating equations logistic regression. History of hospitalisation, history of surgical intervention and history of haematological disease were removed from the final model to reduce collinearity with history of blood transfusion. Examination region was removed to reduce collinearity with WHO region of nationality.
†Children under the age of 15 were excluded from the logistic regression analysis.
*Comparison group

**Table S3.** Testing yield and logistic regression analysis of the syphilis test cohort^†^ (outcome = syphilis positive).

| Variable | Total number screened | Number of positive cases (%) | Unadjusted OR (95% CI) | P value | Adjusted OR (95% CI) | P value |
| --- | --- | --- | --- | --- | --- | --- |
|  |  |  |  |  |  |  |
| **SYPHILIS** | ***n = 9702*** | ***n =23*** |  |  |  |  |
| Age group (years) |  |  |  |  |  |  |
| 15-24* | 2637 | 1 |  |  |  |  |
| 25-34 | 3286 | 4 | 3.21(0.36-28.76) | 0.297 | 3.24(0.33-31.44) | 0.310 |
| 35-49 | 2592 | 13 | **13.29(1.74-101.65)** | 0.013 | **11.97(1.45-99.22)** | 0.021 |
| 50+ | 1108 | 5 | **11.95(1.39-102.40)** | 0.024 | **12.15(1.38-106.65)** | **0.024** |
| Sex |  |  |  |  |  |  |
| Female* | 4995 | 9 |  |  |  |  |
| Male | 4707 | 14 | 1.65(0.71-3.82) | 0.240 | 1.79(0.74-4.35) | 0.198 |
| WHO region of nationality |  |  |  |  |  |  |
| AFR | 960 | 6 | **3.21(1.26-8.17)** | 0.014 | **4.24(1.21-14.82)** | 0.024 |
| EUR | 33 | 0 | No obs |  | No obs |  |
| EMR* | 8705 | 17 |  |  |  |  |
| WPR | 4 | 0 | No obs |  | No obs |  |
| WHO region of examination |  |  |  |  |  |  |
| AFR | 1338 | 3 | 0.86(0.26-2.93) | 0.815 |  |  |
| EUR | 1033 | 1 | 0.37(0.05-2.79) | 0.337 |  |  |
| EMR* | 7331 | 19 |  |  |  |  |
| Year of examination | ***n = 9623*** | ***n = 23*** |  |  |  |  |
| 2013 | 3 | 0 | No obs |  | No obs |  |
| 2014 | 475 | 0 | No obs |  | No obs |  |
| 2015 | 1613 | 2 | **0.24(0.06-1.05)** | 0.058 | **0.15(0.03-0.86)** | 0.033 |
| 2016 | 4413 | 5 | **0.22(0.08-0.60)** | 0.003 | **0.26(0.09-0.72)** | 0.009 |
| 2017* | 3119 | 16 |  |  |  |  |
| HIV positive |  |  |  |  |  |  |
| No* | 9654 | 21 |  |  |  |  |
| Yes | 35 | 2 | **27.80(6.26-123.37)** | <0.001 | **10.27(1.30-81.40)** | 0.027 |
| History of STI |  |  |  |  |  |  |
| No* | 9681 | 18 |  |  |  |  |
| Yes | 21 | 5 | **167.76(55.53-506.77)** | <0.001 |  |  |
| History of torture |  |  |  |  |  |  |
| No* | 8866 | 16 |  |  |  |  |
| Yes | 836 | 7 | **4.67(1.92-11.39)** | 0.001 | **3.27(1.07-9.95)** | 0.037 |
| History of displacement |  |  |  |  |  |  |
| No* | 2407 | 3 |  |  |  |  |
| Yes | 7295 | 20 | 2.20(0.65-7.42) | 0.202 | 1.43(0.36-5.74) | 0.612 |
|  |  |  |  |  |  |  |
| History of tattoos |  |  |  |  |  |  |
| No* | 9160 | 23 |  |  |  |  |
| Yes | 3 | 0 | No obs |  | No obs |  |
| History of blood transfusion |  |  |  |  |  |  |
| No* | 9490 | 23 |  |  |  |  |
| Yes | 212 | 0 | No obs |  | No obs |  |
| History of alcohol intake |  |  |  |  |  |  |
| No* | 9653 | 23 |  |  |  |  |
| Yes | 49 | 0 | No obs |  | No obs |  |
| History of illicit drug use |  |  |  |  |  |  |
| No* | 9676 | 23 |  |  |  |  |
| Yes | 26 | 0 | No obs |  | No obs |  |

OR, odds ratio; CI, confidence interval. Unadjusted and adjusted odds ratios calculated using generalised estimating equations logistic regression. History of STI and examination region were excluded from the final model to reduce collinearity with HIV and WHO region of nationality, respectively.
†Children under the age of 15 were excluded from the logistic regression analysis.
*Comparison group

**Table S4.** Testing yield and logistic regression analysis of the hepatitis B test cohort^†^ (outcome= hepatitis B positive).

| Variable | Total number screened | Number of positive cases (%) | Unadjusted OR (95% CI) | P value | Adjusted OR (95% CI) | P value |
| --- | --- | --- | --- | --- | --- | --- |
|  |  |  |  |  |  |  |
| **HEPATITIS B INFECTION** | ***n = 9228*** | ***n =188*** |  |  |  |  |
| Age group (years) |  |  |  |  |  |  |
| 15-24* | 2484 | 21 |  |  |  |  |
| 25-34 | 3125 | 64 | **2.45(1.49-4.03)** | <0.001 | **2.83(1.69-4.77)** | <0.001 |
| 35-49 | 2463 | 68 | **3.33(2.03-5.45)** | <0.001 | **3.86(2.32-6.41)** | <0.001 |
| 50+ | 1067 | 32 | **3.63(2.08-13.98)** | <0.001 | **4.07(2.34-7.09)** | <0.001 |
| Sex |  |  |  |  |  |  |
| Female* | 4751 | 58 |  |  |  |  |
| Male | 4477 | 130 | **2.42(1.77-3.31)** | <0.001 | **2.66(1.92-3.69)** | <0.001 |
| WHO region of nationality |  |  |  |  |  |  |
| AFR | 854 | 47 | **3.41(2.43-4.78)** | <0.001 | **4.37(2.91-6.55)** | <0.001 |
| EUR | 33 | 1 | 1.83(0.25-13.49) | 0.553 | 3.01(0.45-20.11) | 0.254 |
| EMR* | 8338 | 140 |  |  |  |  |
| WPR | 3 | 0 | No obs |  |  |  |
| WHO region of examination |  |  |  |  |  |  |
| AFR | 1064 | 57 | **3.72(2.68-5.16)** | <0.001 |  |  |
| EUR | 1026 | 24 | 1.57(1.01-2.46) | 0.047 |  |  |
| EMR* | 7138 | 107 |  |  |  |  |
| Year of examination | ***n = 9139*** | ***n = 185*** |  |  |  |  |
| 2014* | 116 | 8 |  |  |  |  |
| 2015 | 1593 | 36 | **0.31(0.14-0.69)** | 0.004 | 0.70(0.30-1.64) | 0.416 |
| 2016 | 4331 | 96 | **0.31(0.15-0.65)** | 0.002 | 0.84(0.37-1.95) | 0.694 |
| 2017 | 3099 | 45 | **0.20(0.01-0.43)** | <0.001 | 0.53(0.23-1.25) | 0.147 |
| HIV positive |  |  |  |  |  |  |
| No* | 9169 | 185 |  |  |  |  |
| Yes | 34 | 2 | 3.04(0.72-12.76) | 0.130 |  |  |
| History of STI |  |  |  |  |  |  |
| No* | 9208 | 184 |  |  |  |  |
| Yes | 20 | 4 | **12.26(4.06-37.03)** | <0.001 | **7.65(2.33-25.18)** | 0.001 |
| History of tattoos |  |  |  |  |  |  |
| No* | 8707 | 174 |  |  |  |  |
| Yes | 521 | 14 | 1.35(0.78-2.35) | 0.282 | 1.07(0.59-1.95) | 0.820 |
| History of blood transfusion |  |  |  |  |  |  |
| No* | 9023 | 179 |  |  |  |  |
| Yes | 205 | 9 | **2.27(1.14-4.50)** | 0.019 | **2.23(1.05-4.76)** | 0.038 |
| History of torture |  |  |  |  |  |  |
| No* | 8414 | 169 |  |  |  |  |
| Yes | 814 | 19 | 1.17(0.72-1.88) | 0.530 | 0.67(0.40-1.14) | 0.138 |
|  |  |  |  |  |  |  |
| History of displacement |  |  |  |  |  |  |
| No* | 2159 | 42 |  |  |  |  |
| Yes | 7069 | 146 | 1.06(0.75-1.50) | 0.730 | 1.15(0.79-1.67) | 0.467 |
|  |  |  |  |  |  |  |
| History of alcohol intake |  |  |  |  |  |  |
| No* | 9179 | 185 |  |  |  |  |
| Yes | 49 | 3 | 3.17(0.98-10.29) | 0.055 | 0.97(0.32-2.89) | 0.950 |
| History of illicit drug use |  |  |  |  |  |  |
| No* | 9205 | 186 |  |  |  |  |
| Yes | 23 | 2 | **4.62(1.08-19.84)** | 0.040 | 1.50(0.21-10.52) | 0.681 |
| History of TB |  |  |  |  |  |  |
| No* | 9205 | 187 |  |  |  |  |
| Yes | 23 | 1 | 2.19(0.29-16.35) | 0.444 | 0.98(0.10-9.74) | 0.983 |

OR, odds ratio; CI, confidence interval.
Unadjusted and adjusted odds ratios calculated using generalised estimating equations logistic regression. HIV and examination region were excluded from the final model to reduce collinearity with history of STI and WHO region of nationality, respectively. No data for 2013.
†Children under the age of 15 were excluded from the logistic regression analysis.
*Comparison group

**Table S5.** Testing yield and logistic regression analysis of the hepatitis C test cohort^†^ (outcome: hepatitis C positive).

| Variable | Total number screened | Number of positive cases (%) | Unadjusted OR (95% CI) | P value | Adjusted OR (95% CI) | P value |
| --- | --- | --- | --- | --- | --- | --- |
|  |  |  |  |  |  |  |
| **HEPATITIS C INFECTION** | ***n = 9223*** | ***n =38*** |  |  |  |  |
| Age group (years) |  |  |  |  |  |  |
| 15-24* | 2483 | 6 |  |  |  |  |
| 25-34 | 3123 | 8 | 1.06(0.37-3.51) | 0.914 | 1.07(0.37-3.06) | 0.898 |
| 35-49 | 2461 | 7 | 1.18(0.40-3.51) | 0.769 | 1.21(0.41-3.60) | 0.732 |
| 50+ | 1067 | 17 | **6.68(2.63-17.00)** | <0.001 | **6.71(2.67-16.87)** | <0.001 |
| Sex |  |  |  |  |  |  |
| Female* | 4746 | 19 |  |  |  |  |
| Male | 4477 | 19 | 1.06(0.56-2.01) | 0.857 | 1.16(0.62-2.19) | 0.634 |
| WHO region of nationality |  |  |  |  |  |  |
| AFR | 853 | 5 | 1.48(0.58-3.81) | 0.413 | 2.39(0.91-6.33) | 0.077 |
| EUR | 33 | 0 | No obs |  | No obs |  |
| EMR* | 8334 | 33 |  |  |  |  |
| WPR | 3 | 0 | No obs |  | No obs |  |
| WHO region of examination |  |  |  |  |  |  |
| AFR | 1061 | 5 | 1.40(0.53-3.69) | 0.492 |  |  |
| EUR | 1025 | 9 | **2.63(1.22-5.66)** | 0.014 |  |  |
| EMR* | 7137 | 24 |  |  |  |  |
| Year of examination | ***n = 9803*** | ***n = 23*** |  |  |  |  |
| 2014 | 116 | 0 | No obs |  | No obs |  |
| 2015 | 1590 | 3 | 0.58(0.16-2.12) | 0.414 | 0.47(0.13-1.79) | 0.271 |
| 2016 | 4329 | 25 | 1.79(0.86-3.74) | 0.119 | 1.66(0.76-3.65) | 0.207 |
| 2017* | 3099 | 10 |  |  |  |  |
| HIV positive |  |  |  |  |  |  |
| No* | 9165 | 38 |  |  |  |  |
| Yes | 34 | 0 | No obs |  |  |  |
| History of STI |  |  |  |  |  |  |
| No* | 9203 | 38 |  |  |  |  |
| Yes | 20 | 0 | No obs |  | No obs |  |
| History of tattoos |  |  |  |  |  |  |
| No* | 8702 | 35 |  |  |  |  |
| Yes | 521 | 3 | 1.43(0.44-4.68) | 0.550 | 1.79(0.55-5.79) | 0.333 |
| History of blood transfusion |  |  |  |  |  |  |
| No* | 9018 | 34 |  |  |  |  |
| Yes | 205 | 4 | **5.26(1.85-14.96)** | 0.002 | **5.19(1.70-15.88)** | 0.004 |
| History of torture |  |  |  |  |  |  |
| No* | 8409 | 36 |  |  |  |  |
| Yes | 814 | 2 | 0.57(0.14-2.38) | 0.444 | 0.63(0.15-2.70) | 0.533 |
|  |  |  |  |  |  |  |
| History of displacement |  |  |  |  |  |  |
| No* | 2158 | 11 |  |  |  |  |
| Yes | 7065 | 27 | 0.75(0.37-1.51) | 0.420 | 0.77(0.39-1.55) | 0.471 |
|  |  |  |  |  |  |  |
| History of alcohol intake |  |  |  |  |  |  |
| No* | 9174 | 38 |  |  |  |  |
| Yes | 49 | 0 | No obs |  | No obs |  |
|  |  |  |  |  |  |  |
| History of illicit drug use |  |  |  |  |  |  |
| No* | 9200 | 38 |  |  |  |  |
| Yes | 23 | 0 | No obs |  | No obs |  |
| History of TB |  |  |  |  |  |  |
| No* | 9200 | 38 |  |  |  |  |
| Yes | 23 | 0 | No obs |  | No obs |  |

OR, odds ratio; CI, confidence interval.
Unadjusted and adjusted odds ratios calculated using generalised estimating equations logistic regression. HIV and examination region were excluded from the final model to reduce collinearity with history of STI and WHO region of nationality, respectively. No data for 2013.
†Children under the age of 15 were excluded from the logistic regression analysis.
*Comparison group
